# Effects and formula optimization of *Rosa roxbunghii* pomace substrate on the yield and volatile flavor compounds of *Lentinulaedodes*

**DOI:** 10.3389/fpls.2025.1456290

**Published:** 2025-05-26

**Authors:** Tingfei Deng, Liangqun Li, Chunyan Li, Yanfang Yan, Anqin Gao, Xiaolan Liu, Xiong Pan, Ming Gao, Lijuan Ge, Mei Peng, Zhongsheng Luo, Yan Tian, Juan Yang, Xiaosheng Yang

**Affiliations:** ^1^ State Key Laboratory of Discovery and Utilization of Functional Components in Traditional Chinese Medicine, Guizhou Medical University, Guiyang, China; ^2^ Natural Products Research Center of Guizhou Province, Guiyang, China; ^3^ Liupanshui Agricultural and Rural Bureau of Guizhou Province, Liupanshui, China; ^4^ Wengfu (Group) Co., Ltd, Guiyang, China

**Keywords:** *Rosa roxbunghii* pomace, Lentinus edodes, volatile flavor components, gas chromatography-mass spectrometry, principal components analysis

## Abstract

**Introduction:**

*Rosa roxbunghii* pomace (RRP), a by-product of R. roxbunghii processing, remains largely underutilized. Given the increasing demand for sustainable and resource-efficient mushroom cultivation methods, exploring RRP as a mushroom cultivation medium could not only address waste management issues but also potentially enhance the quality of cultivated mushrooms. This study aimed to investigate the effects of RRP at different proportions on the yield and volatile flavor compounds of Lentinula edodes, and to optimize the formula of the cultivation substrate.

**Methods:**

Different proportions of RRP were incorporated into the cultivation substrate of Lentinula edodes to form various test formulas. The mushroom yield of each formula was measured by counting the harvested weight per cultivation bag. Advanced gas chromatography-mass spectrometry (GC-MS) was employed to analyze the volatile flavor profiles of the mushrooms cultivated under different formulas. Principal component analysis (PCA) was conducted to explore the differences in volatile flavor substances among formulas and compared with the control group.

**Results:**

Among the tested formulas, a mixture containing 30% RRP (Formula 2) showed the highest yield, reaching 0.85 kg per cultivation bag, significantly outperforming other formulas. GC-MS analysis revealed distinct volatile flavor profiles for each formula. Formula 2 was characterized by its unique flavor attributes, with 1-octen-3-ol accounting for 29.16% of the relative content, a key compound contributing to the umami flavor of mushrooms. PCA results further confirmed that the volatile flavor substances of Formula 2 were significantly different from those of the control group.

**Discussion:**

These findings demonstrate that incorporating RRP into mushroom cultivation substrates can enhance both yield and flavor characteristics of L. edodes. The optimal formulation (30% RRP) not only supported maximum productivity but also contributed to a more desirable aroma profile. This study presents an innovative and sustainable approach to repurposing agricultural waste, adding value to both mushroom production and resource utilization.

## Graphical Abstract

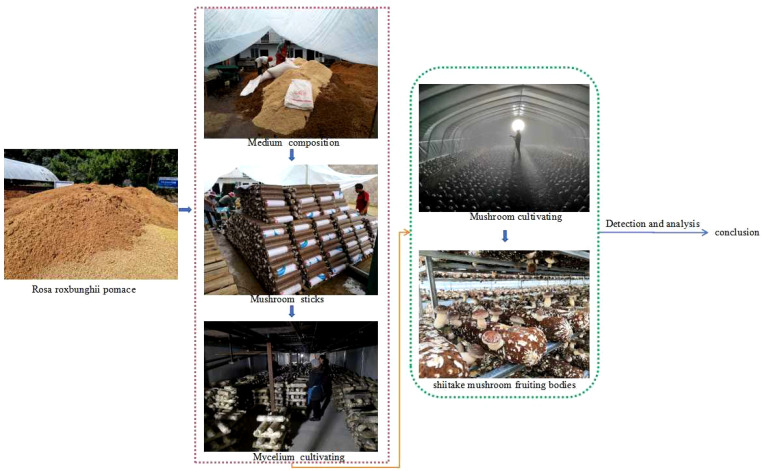

## 1 Introduction

The edible fungus industry in Guizhou province, China, promotes farmers’ income, agricultural efficiency, and rural development. Shiitake mushrooms (Lentinula edodes), rich in nutrients like high-protein, low-fat, polysaccharides, amino acids, vitamins, and trace elements, also possess a delicious taste and intense aroma, having significant medicinal and edible value (Xu and Liu, 1987; Lu and Chen, 2004; Cao et al., 2017). In 2021, Guizhou province’s edible fungus planting scale, production, and output value were expected to experience substantial growth.

Research on mushroom aroma has been ongoing. Many studies have focused on identifying and analyzing volatile flavor compounds in mushrooms. For example, previous research has shown that octanes and sulfur-containing compounds are the main components contributing to the unique aroma of shiitake mushrooms (An et al., 2012). Understanding these aroma-related substances can help improve the quality of cultivated mushrooms.

Guizhou’s climate is suitable for the large-scale cultivation of *Rosa roxbunghii*, which has a large-scale cultivation area and high fruit production. The province’s *Rosa roxbunghii* cultivation area has reached 140,000 hectares, and the fresh fruit production has reached 130,000 tons. However, due to the limited consumption capacity of fresh *Rosa roxbunghiis*, with a large amount of *Rosa roxbunghii* cultivation entering the fruiting period, the purchasing pressure of processing enterprises is relatively high, and they mainly choose to squeeze juice for preservation. Consequently, the resulting RRP becomes waste, causing significant resource waste and serious environmental problems.

Long-term improper stacking and abandonment of plant pomaces such as RRP can not only waste resources but also generate ammonia (Watanabe et al., 2011) and release ammonia ions. After some ammonia is emitted, it contains harmful exhaust gases, which enter the atmosphere and cause severe air pollution. Due to the high content of woody fibers in RRP (Shi et al., 2006), the degradation cycle is long, and the harmful substances produced can flow into rivers and lakes with natural precipitation, causing severe surface water pollution.

Currently, RRP is mainly used in soluble dietary fiber research, gastrointestinal flora improvement, and animal feeding (Qin et al., 2021; Tian et al., 2021; Wang et al., 2021; Zhang and Tian, 1996; Zheng and Huang, 1996; Cheng et al., 1988), but its application in shiitake mushroom cultivation remains unreported.

Notably, the interaction between mushrooms and plants, such as mycorrhizal symbiosis, plays a vital role in the growth and development of both parties. A study has demonstrated that mycorrhizal symbiosis can alter plants’ composition of secondary metabolites (Rashidi et al., 2024). In the context of mushroom cultivation, this kind of interaction might affect the nutrient absorption of mushrooms and the synthesis of their volatile flavor compounds (Yang et al., 2019; Zhou et al., 2022). For example, the symbiotic relationship could influence the availability of nutrients like nitrogen and phosphorus to mushrooms, which may impact the metabolic pathways related to the production of flavor-related substances. Understanding such interactions is crucial for optimizing mushroom cultivation substrates and improving the quality of cultivated mushrooms. This study aims to explore using RRP as a substrate for shiitake mushrooms as a control cultivation, comparing its effects on yield and volatile flavor compounds with traditional sawdust-based cultivation to provide new insights into the edible mushroom and *Rosa roxbunghii* industries.

## 2 Materials and methods

### 2.1 Materials and reagents

Shiitake mushroom strain: variety 238; Herbarium number: GZMU-202308. *Rosa roxbunghii* pomace: provided by Liupanshui Chunhao Agricultural Science and Technology Development Co., Ltd.; sawdust: mixed sawdust purchased from the market.

### 2.2 Experimental methods and experimental design

Four shiitake mushroom substrate formulations (see **Table 1**) were used in the experiment, with 3 replicates and 20 bags per replicate for 60 bags per formulation. The experiment was conducted at the Key Laboratory of Natural Product Chemistry of the Chinese Academy of Sciences in Guizhou Province.

**Table 1:** Medium composition of Lentinus edodes.

Number	Medium composition
1	69% sawdust+1% gypsum+10% R. roxburghii pomace +20% lumpy wheat bran
2	49% sawdust +1% gypsum +30% R. roxburghii pomace +20% lumpy wheat bran
3	29% sawdust +1% gypsum +50% R. roxburghii pomace +20% lumpy wheat bran
control group	79% sawdust +1% gypsum +20% lumpy wheat bran

### 2.3 Bag preparation

The ingredients were weighed according to the formulations in **Table 1**, mixed with water to a moisture content of 45-50%, and packed into 15x55cm low-pressure polyethylene bags. Each bag contained 1300g of dry material and was sterilized by conventional atmospheric pressure sterilization. After cooling, 3 holes were made in each bag and inoculated, and then the bags were incubated at a constant temperature of 25°C.

### 2.4 Mushroom management

After the mycelium reached physiological maturity, holes were made using a special shiitake mushroom punching machine. Then, the bags were stacked in a cross shape, with air circulation and timely heat dissipation. Following the observable mycelial chromatic transition, the cultivation bags were aseptically transferred to designated shelving units to initiate the fructification phase. After a 7-day incubation period, primordia differentiation became evident, during which environmental parameters were rigorously maintained: temperature regulated at 15–25°C (optimal range for basidiocarp development), relative humidity sustained at 85–90% RH, and atmospheric gas exchange ensured through automated climate control systems supplemented with periodic forced-air ventilation. After each crop was harvested, the mushroom sticks were allowed to rest for 7-10 days to allow the mycelium to recover before being watered and cultivated again.

### 2.5 Instruments and equipment

HP6890/5975C GC/MS system (Agilent Technologies, USA), mixer, packaging machine, bagging machine, and punching machine.

### 2.6 Methods

#### 2.6.1 Production of shiitake mushroom fruiting bodies

Mixed sawdust purchased from the market was used, and different ratios of RRP were added to the shiitake mushroom cultivation. The shiitake mushrooms were harvested when they had just sprouted and were tested within 12 hours. The substrate formulations are shown in **Table 1**.

#### 2.6.2 Sample preparation

Weigh approximately 4g of mixed and uniformed shiitake mushroom samples, place them in a solid-phase microextraction sampling bottle, insert a manually operated syringe with a 2 cm-50/30μm DVB/CAR/PDMS StableFlex fiber head, and perform headspace extraction for 60 minutes under the condition of 60°C flat heating. After removing the extraction head, insert it into the gas chromatograph inlet (temperature 250°C) and perform thermal analysis.

#### 2.6.3 Chromatographic conditions

The chromatographic column is an Agilent 19091S-436HP-5MS (60m × 250μm × 0.25μm) flexible quartz capillary column. The initial temperature is 40°C (hold for 2 minutes), and then the temperature is increased at a rate of 3.5°C/min to 208°C, followed by an increase of 10°C/min to 308°C. The total run time is 60 minutes. The temperature of the vaporization chamber is 250°C. High-purity helium (99.999%) is used as the carrier gas. The column head pressure is 15.85psi, the carrier gas flow rate is 1.0 mL/min, and the split ratio is 5:1. The solvent delay time is 3 minutes. When calculating the retention indices of volatile substances, n-alkanes (C8 - C20) are used as reference standards for calculating retention indices.

#### 2.6.4 Mass spectrometry conditions

The ion source is an EI source, with an ion source temperature of 230°C, a quadrupole temperature of 150°C, an electron energy of 70eV, an emission current of 34.6 μ4, a multiplier voltage of 1623V, an interface temperature of 280°C, and a mass range of 29~500amu.

#### 2.6.5 Data analysis

The gas chromatography-mass spectrometry (GC-MS) analysis spectrum is matched and searched with the NIST Library and Wiley Library for each peak through computer and manual calculation to determine the qualitative composition of each component in the sample (the percentage of component peak area to the total peak area) according to the normalization method of peak area. Principal component analysis (PCA) is conducted using SIMCA14.1 software.

## 3 Results

### 3.1 Effects of different additive ratios on shiitake mushroom yield and benefits in cultivation

The yield, moisture, and ash content of shiitake mushrooms cultivated with different additive ratios of *Rosa roxbunghii* pomace are shown in **Table 2**. It can be seen that there is no significant difference in the number of fruiting bodies and yield between formula 2 and the control. In contrast, formula 2, formula 1, and formula 3 show significant differences in yield. The moisture and ash content of shiitake mushrooms produced with different additive ratios all meet the GB/T 38581-2020 standard. From the economic benefits, it can be seen that using formula 2 to replace sawdust alleviates the demand for sawdust to a certain extent, reasonably disposes of *Rosa roxbunghii* pomace, reduces raw material costs, and is suitable for the cultivation of shiitake mushrooms using *Rosa roxbunghii* pomace as an additive in Guizhou region.

**Table 2:** Results of lentinus edodes with different proportion of cultivation materials(x ± s,n=3).

Formulation number	Inoculation date (Month -Day)	Mushroom production date/d	Yield(g/bar)	Number of mushroom stubbles	Moisture content/%	Ash content/%
1	02-02	06-01	600 ± 30bB	5	86.7 ± 0.34bB	6.0 ± 0.4cBC
2	02-02	06-01	850 ± 20aA	6	89.9 ± 0.20aA	7.8 ± 0.04aA
3	02-02	06-01	550 ± 40bB	4	87.0 ± 0.35bB	6.8 ± 0.3bB
control group	02-02	06-01	800 ± 20aA	6	83.2 ± 0.17cC	5.3 ± 0.2dC

a, b differ significantly (p < 0.05); A, B differ highly significantly (p < 0.01).

### 3.2 Effects of different ratios of added RRP on the types and contents of volatile compounds in cultivated shiitake mushrooms

The volatile flavor compounds and relative contents of shiitake mushrooms grown with different ratios of added RRP are shown in **Tables 3**, **4**. A total of 50 compounds were detected in the four formulations, with 4 compounds remaining unidentified. Among them were 10 hydrocarbons, 15 alcohols, 13 aldehydes, 5 ketones, 1 ether, 1 lipid, 1 aromatic compound, and 4 other substances. Formulations 1, 2, 3, and the control contained 50, 45, 48, and 44 volatile compounds, respectively. Formulation 1 had the highest number of volatile compounds. In formulations 1 and 3, ketones were the most abundant compounds, followed by alcohols and aldehydes. In the control, ketones were also the most abundant, followed by alcohols and other compounds. This suggests that formulations 1 and 3 differ somewhat from the control regarding flavor. Formulation 2, on the other hand, had the highest content of alcohols, followed by ketones and then aldehydes. This indicates that formulation 2 differs significantly from formulations 1 and 3, and the control suggests that adding 30% RRP substantially impacted the volatile compound content of shiitake mushrooms. This may be due to the influence of different proportions of mushroom strains on the matrix formula content.

**Table 3:** Volatile flavor components and relative contents of Lentinus edodes cultivated with *Rosa roxbunghii* pomace in different supplemental proportions(x ± s,n=3).

Compound name	Retention time/min	Retention index	Percentage of components in each formula sample/%				VIP value
			1	2	3	Control group	
acetaldehyde	4.15	404	0.013 ± 0.003	0.055 ± 0.001	0.031 ± 0.011	0.010 ± 0.003	0.98
Methyl mercaptan	4.265	401	0.039 ± 0.014	–	0.058 ± 0.001	0.133 ± 0.035	0.89
ethanol	4.356	427	0.248 ± 0.001	1.235 ± 0.049	0.658 ± 0.02	0.189 ± 0.01	1.01
acetone	4.575	486	0.012 ± 0.003	–	0.012 ± 0.004	0.032 ± 0.006	0.91
carbon disulfide	4.926	549	4.787 ± 0.025	3.946 ± 0.045	5.348 ± 0.027	12.150 ± 0.027	0.96
Isovaleraldehyde	6.5	652	0.047 ± 0.002	0.061 ± 0.005	0.021 ± 0.008	0.022 ± 0.009	0.94
2-Methylbutyraldehyde	6.701	662	0.018 ± 0.001	0.024 ± 0.009	0.007 ± 0.001	0.010 ± 0.002	0.88
Valeraldehyde	7.471	699	0.008 ± 0.004	0.010 ± 0.004	0.007 ± 0.001	0.057 ± 0.017	0.95
Butyl formate	8.235	724	0.011 ± 0.004	–	–	–	1.10
Isoamyl alcohol	8.526	736	0.037 ± 0.01	0.065 ± 0.01	0.058 ± 0.01	0.025 ± 0.004	0.94
2-Methylbutanol	8.634	739	0.022 ± 0.009	0.049 ± 0.01	0.030 ± 0.005	0.020 ± 0.007	0.89
Dimethyl disulfide	8.865	746	0.351 ± 0.008	0.559 ± 0.05	0.722 ± 0.036	2.252 ± 0.01	0.98
toluene	9.612	763	0.011 ± 0.002	0.029 ± 0.006	0.021 ± 0.001	–	0.96
1-pentanol	9.636	765	0.043 ± 0.004	0.033 ± 0.003	0.056 ± 0.003	–	1.05
1-octene	10.352	789	0.023 ± 0.004	0.034 ± 0.004	0.014 ± 0.003	0.014 ± 0.005	0.91
Hexanal	10.751	800	0.032 ± 0.005	0.013 ± 0.003	0.007 ± 0.001	–	1.07
1,3-Octadiene	11.708	827	0.070 ± 0.02	0.062 ± 0.009	0.040 ± 0.01	0.021 ± 0.006	0.88
N-hexanol	13.65	868	0.044 ± 0.005	0.020 ± 0.001	0.018 ± 0.003	–	1.07
3-Heptanone	14.337	887	0.008 ± 0.001	0.008 ± 0.002	0.018 ± 0.003	0.011 ± 0.006	0.93
Oxime-, methoxy-phenyl-_	15.412		0.182 ± 0.015	0.286 ± 0.01	0.407 ± 0.05	0.007 ± 0.001	1.05
Benzaldehyde	17.73	962	5.489 ± 0.017	3.884 ± 0.01	6.670 ± 0.04	2.512 ± 0.009	1.10
Dimethyl trisulfide	18.164	970	0.682 ± 0.017	0.790 ± 0.03	1.309 ± 0.019	3.124 ± 0.02	0.97
1-Octene-3-one	18.449	979	7.581 ± 0.01	7.125 ± 0.02	1.851 ± 0.02	2.642 ± 0.036	1.04
1-Octene-3-ol	18.748	980	19.491 ± 0.046	29.160 ± 0.02	6.116 ± 0.032	9.143 ± 0.01	0.99
3-Octanone	18.955	986	36.044 ± 0.01	28.762 ± 0.02	46.045 ± 0.02	32.957 ± 0.03	1.11
3-octanol	19.302	994	11.141 ± 0.015	8.649 ± 0.02	12.511 ± 0.026	11.604 ± 0.02	1.02
n-octanal	19.565	1003	0.307 ± 0.02	0.519 ± 0.006	0.331 ± 0.02	0.133 ± 0.007	0.94
2-ethylhexyl alcohol	20.727	1030	0.215 ± 0.008	0.087 ± 0.002	0.090 ± 0.008	0.026 ± 0.004	1.10
Benzyl alcohol	21.337	1036	0.039 ± 0.002	0.049 ± 0.003	0.107 ± 0.006	0.038 ± 0.004	1.13
Phenylacetaldehyde	21.539	1045	0.350 ± 0.026	0.373 ± 0.021	0.338 ± 0.01	0.144 ± 0.012	0.95
Trans-2-octenal	22.047	1060	0.513 ± 0.012	1.084 ± 0.005	0.401 ± 0.011	0.066 ± 0.006	0.94
Trans-2-octene-1-ol	22.478	1067	0.691 ± 0.089	2.563 ± 0.038	0.596 ± 0.015	0.167 ± 0.01	0.97
Octanol	22.578	1071	3.219 ± 0.009	4.722 ± 0.009	4.483 ± 0.01	2.029 ± 0.03	1.01
Nonyl aldehyde	24.085	1104	0.050 ± 0.002	0.027 ± 0.002	0.035 ± 0.006	0.064 ± 0.004	0.97
2-Phenylpropionaldehyde	24.182	1094	0.103 ± 0.003	0.095 ± 0.003	0.195 ± 0.003	0.154 ± 0.004	1.06
Phenylethanol	24.733	1116	0.385 ± 0.004	0.577 ± 0.007	0.605 ± 0.008	0.322 ± 0.02	1.06
2,3,5-Trithiahexane	25.267	1143	0.159 ± 0.015	0.217 ± 0.019	0.402 ± 0.003	0.769 ± 0.01	0.97
2-Phenylpropenal	26.535	1150	0.799 ± 0.036	0.421 ± 0.019	0.932 ± 0.019	1.334 ± 0.019	0.92
1-Nonanol	26.978	1173	0.145 ± 0.017	–	0.124 ± 0.006	0.102 ± 0.012	1.06
2-phenyl-1-propanol	27.26	1179	0.107 ± 0.025	0.073 ± 0.006	0.338 ± 0.008	0.351 ± 0.013	0.99
Dodecane	28.095	1200	0.013 ± 0.003	0.009 ± 0.001	0.018 ± 0.002	0.013 ± 0.013	0.98
Decanal	28.423	1206	0.029 ± 0.004	–	–	–	1.14
Dimethyl tetrasulfide	29.191	1234	0.114 ± 0.009	0.135 ± 0.01	0.392 ± 0.016	1.901 ± 0.019	0.97
Methyl nonyl ketone	31.98	1294	0.042 ± 0.003	0.015 ± 0.003	0.031 ± 0.007	0.027 ± 0.005	1.04
Tridecane	32.145	1300	0.023 ± 0.005	0.012 ± 0.003	0.030 ± 0.001	0.028 ± 0.004	0.92
1,2,4,5-tetrathiocyclohexane	34.526	1337	0.735 ± 0.017	0.501 ± 0.009	0.581 ± 0.005	1.667 ± 0.012	0.97
Tetradecane	35.965	1400	0.025 ± 0.001	0.007 ± 0.002	0.015 ± 0.005	0.020 ± 0.01	0.9
1,2,4,6-Tetrathiepane	40.724	1483	0.123 ± 0.01	0.042 ± 0.003	0.172 ± 0.003	0.166 ± 0.005	0.98
1-Hexadecene	42.98	1600	0.021 ± 0.004	0.004 ± 0.001	0.010 ± 0.007	0.009 ± 0.003	1.01
Lenthionine	44.691	1603	0.721 ± 0.015	0.343 ± 0.005	1.152 ± 0.005	3.309 ± 0.032	0.95

“-” means not detected.

**Table 4:** Species and content of volatile flavor components of Lentinus edodes cultivated with *RRP* in different supplemental proportions(n=3).

Formula	Hydrocarbons		Alcohols		Aldehydes		Ketones		Ethers		Esters		Aromatic compounds		Others	
	Type	Content/%	Type	Content/%	Type	Content/%	Type	Content/%	Type	Content/%	Type	Content/%	Type	Content/%	Type	Content/%
1	10	1.91	15	35.87	13	7.76	5	43.69	1	0.11	1	0.01	1	0.011	4	6.01
2	10	1.228	12	47.28	12	6.57	4	35.91	1	0.14	0	0	1	0.029	4	5.58
3	10	2.437	15	25.85	12	8.98	5	47.96	1	0.39	0	0	1	0.021	4	7.79
control group	10	6.013	13	24.15	11	4.51	5	35.67	1	1.9	0	0	0	0	4	17.53

Compared with the results of Liang et al (Liang et al., 2020; Chen et al., 2018; MAGA, 1981), The differences in the content of volatile compounds were relatively significant, which may be due to the different substrates used for shiitake mushroom cultivation.

As shown in **Table 3**, 19 compounds exhibited VIP values greater than 1. Notably, Decanal (VIP = 1.14271), Benzyl alcohol (VIP = 1.1322), 3-octanone (VIP = 1.11163), Benzaldehyde (VIP = 1.10324), and Hexanal (VIP = 1.07226) demonstrated significant contributions to the model’s discrimination between experimental groups, suggesting their potential as key biomarkers for inter-group differentiation.

The odor of shiitake mushrooms is mainly composed of two types of volatile compounds: octanes and sulfur-containing compounds. Other volatile compounds such as aldehydes, lipids, ketones, and alcohols play a role in adjusting and enhancing the fragrance. In contrast, phenols, acids, hydrocarbons, and other substances have less of an impact on the characteristic odor of shiitake mushrooms (An et al., 2012). Octanes primarily refer to octane and its derivatives, which are the main fragrance components of fresh shiitake mushrooms. The most representative ones are 1-octen-3-ol, 3-octanol, and 3-octanoate (Huang, 2012). The relative contents of formulas 1, 2, 3, and the control were 66.68%, 66.57%, 64.67%, and 53.7%, respectively. 1-octen-3-ol, also known as mushroom alcohol, is the most characteristic substance with a fresh wild mushroom odor. The highest relative content of 1-octen-3-ol was found in formula 2 at 29.16%, followed by formula 1 at 19.49%, the control at 9.14%, and the lowest was formula 3 at 6.12%.

Sulfur-containing compounds are typical aromatic substances and an important source of the aroma of shiitake mushrooms, which can affect the overall odor of shiitake mushrooms(Stefan Schulz et al., 2010). The content of sulfur-containing compounds in formula 1 was 4.87%, in formula 2 was 3.36%, in formula 3 was 6.22%, and in the control was 11.03%. The highest dimethyl disulfide and dimethyl trisulfide content was found in the control, at 2.25% and 3.12%, respectively, and the lowest content was found in formula 1, at 0.35% and 0.68%, respectively. The differences in the dimethyl disulfide and dimethyl trisulfide contents between different formulas indicated a significant difference in the volatile flavor of shiitake mushrooms grown with varying proportions of RRP. Dimethyl tetrasulfide is rarely reported in the literature on the flavor of shiitake mushrooms. Huang Wen believed that dimethyl tetrasulfide is only produced during the drying process of shiitake mushrooms and is not detected in fresh shiitake mushrooms. In this study, dimethyl tetrasulfide was not detected in any of the four mushroom formulas.

### 3.3 Principal component analysis of volatile flavor components in cultivated shiitake mushroom with different adding ratios of RRP

**Figure 1:**
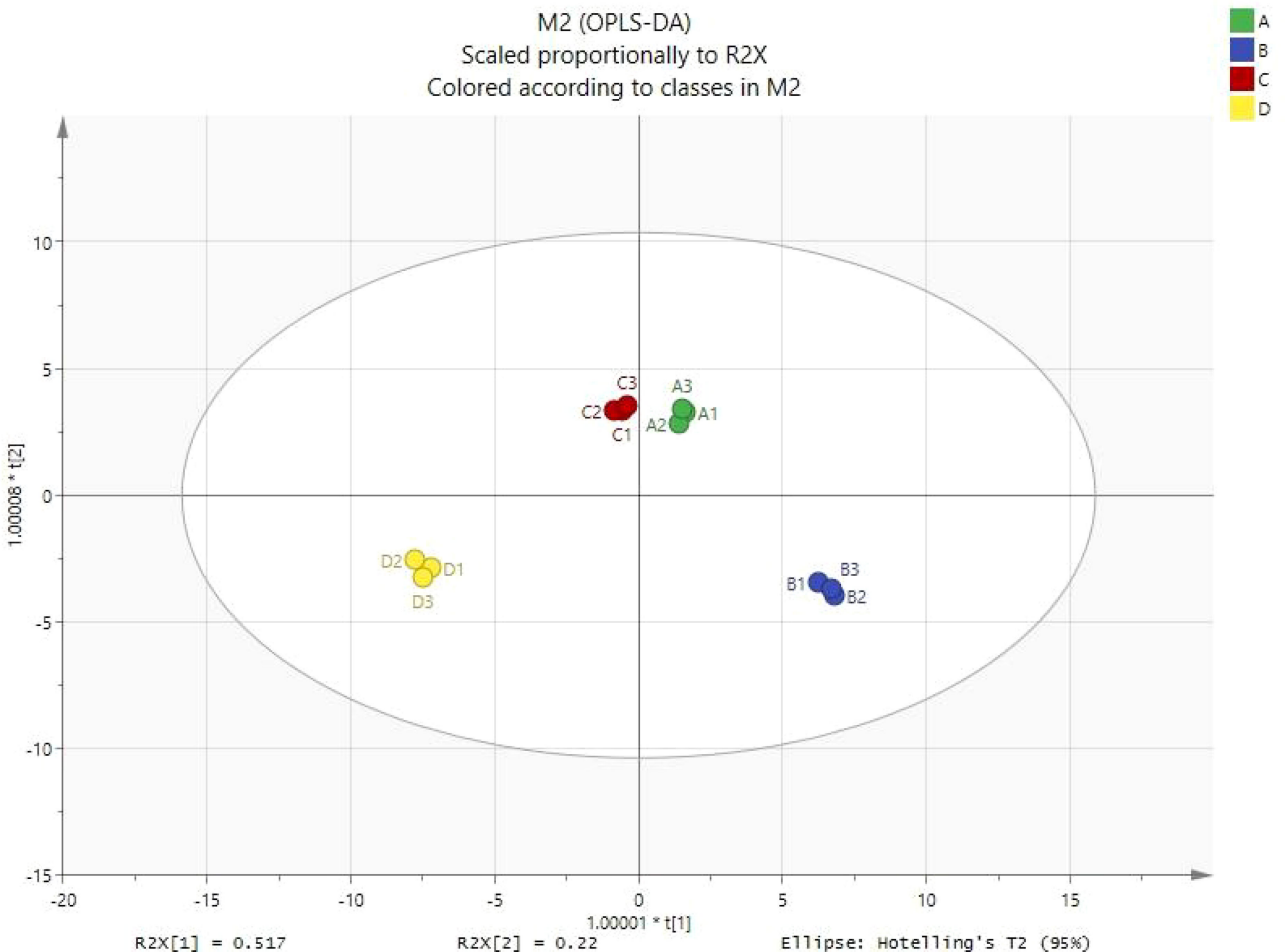
OPLS-DA score chart (A1 formula 1; B1 formula 2; C1 formula 3; D1 the control group, the same below).

Shiitake mushrooms contain numerous volatile components. Principal component analysis (OPLS-DA) was used to distinguish the differences in volatile components among four types of shiitake mushrooms and to understand which components play a significant role. As shown in **Figure 1**, Formula 2 is far from the control and is located in the diagonal position, indicating a significant difference in volatile flavor substances between Formula 2 (with 30% added litchi waste pomace as the culture medium) and the control (sawdust culture medium). Formula 1 and Formula 3 are distributed in different quadrants but are relatively close, indicating a similar composition of volatile components in these two formulas.

**Figure 2:**
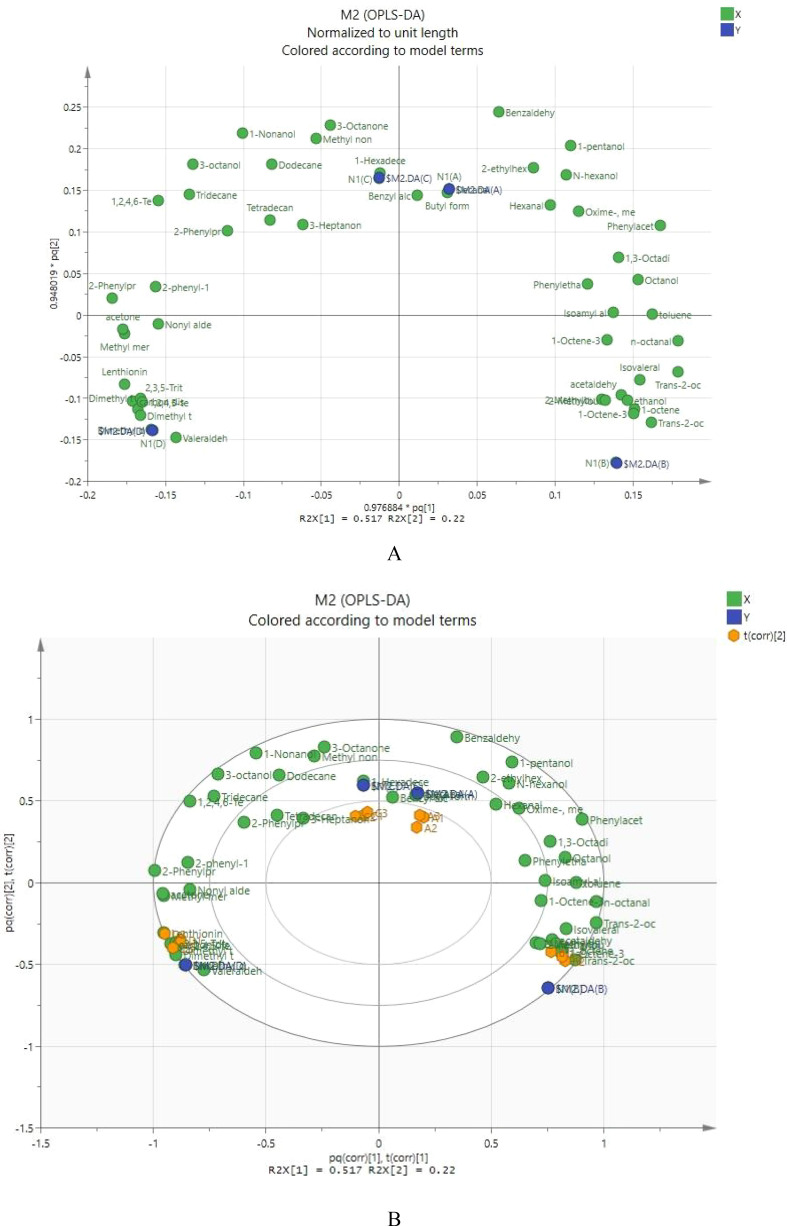
**(A)** OPLS-DA loading plot **(B)** Correlation of volatile substances in Lentinus edodes.

In **Figure 2A**, the horizontal axis represents the first principal component, and the points farthest from the horizontal axis significantly influence the first principal component. The vertical axis represents the second principal component, and the points farthest from the vertical axis significantly affect the second principal component. In this study, the most significant contributions to the first principal component were made by trans-2-octenal and octanal, followed by benzaldehyde, toluene, 1,3-octadiene, trans-2-octen-1-ol, isovaleraldehyde, and 1-octene. The most significant contributions to the second principal component were made by benzaldehyde, followed by 3-octanone, 1-pentanol, and dodecane.

In **Figure 2B**, Formula 1, Formula 2, Formula 3, and the control are distributed in different quadrants. Formula 1 is located in the first quadrant and shows a positive distribution on the first and second principal components. Volatile components such as 1,3-octadiene, toluene, benzaldehyde, benzyl alcohol, 1-pentanol, isovaleric acid, and 2-ethylhexanol showed a positive correlation with Formula 1. Formula 3 is located in the second quadrant and shows a negative correlation with the first principal component and a positive correlation with the second principal component. Volatile components such as 3-octanol, 3-octanone, dodecane, 1-2-4-6-tetrathia-hexane, and 2-phenylacetaldehyde are primarily present in Formula 3. The control is located in the third quadrant, showing a negative correlation with both the first and second principal components. Volatile components such as methanethiol, acetone, dimethyl disulfide, valeraldehyde, dimethyl tetrasulfide, and ergosterol are the main volatile components of the control. Formula 2 is located in the fourth quadrant, showing a positive correlation with the first principal component and a negative correlation with the second principal component. Volatile components such as 1-octen-3-ol, trans-2-octen-1-ol, trans-2-octenal, 2-methyl-1-butanol, 2-methylbutanal, and 1-octene are primarily present in Formula 2.

**Figure 3:**
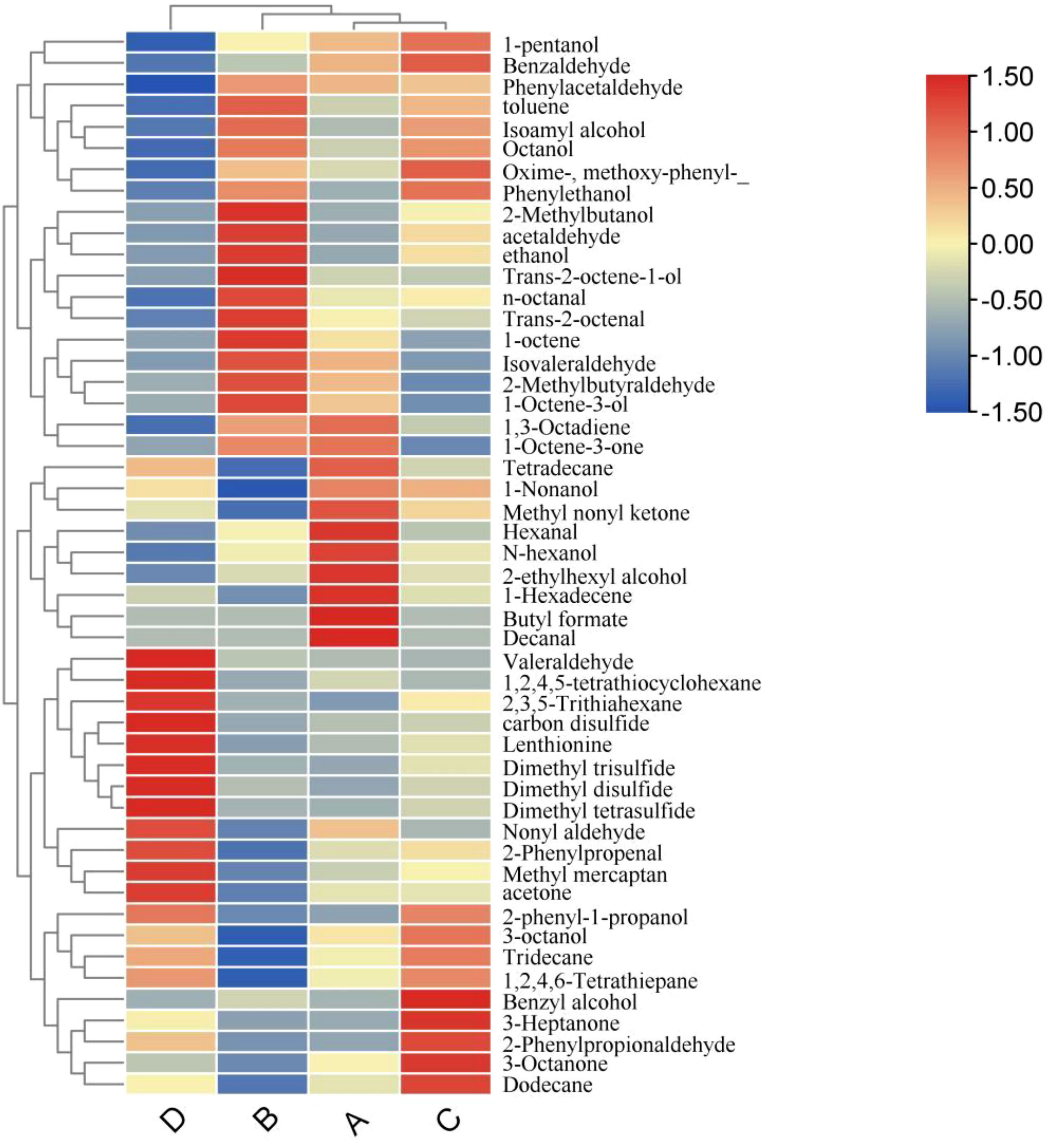
Cluster heatmap [**(A)** formula 1; **(B)** formula 2; **(C)** formula 3; **(D)** the control group].

**Figure 3** illustrates the compositional analysis reveals the following metabolic profiles across experimental groups: Formula 1 contains 50 compounds, Formula 2 comprises 45 compounds, Formula 3 includes 48 compounds, and Control Group comprises 44 compounds. Pairwise intersections demonstrate: Formula 1 & Formula 2 share 45 overlapping compounds, Formula 1 & Formula 3 exhibit 48 shared compounds, Formula 1 & Control Group have 44 common compounds, Formula 2 & Formula 3 show 45 co-occurring compounds, Formula 2 & Control Group overlap with 41 shared compounds, Formula 3 & Control Group share 44 compounds. Notably, 41 compounds are universally conserved across all four groups, representing a core metabolomic signature indicative of fundamental biochemical processes.

**Figure 4:**
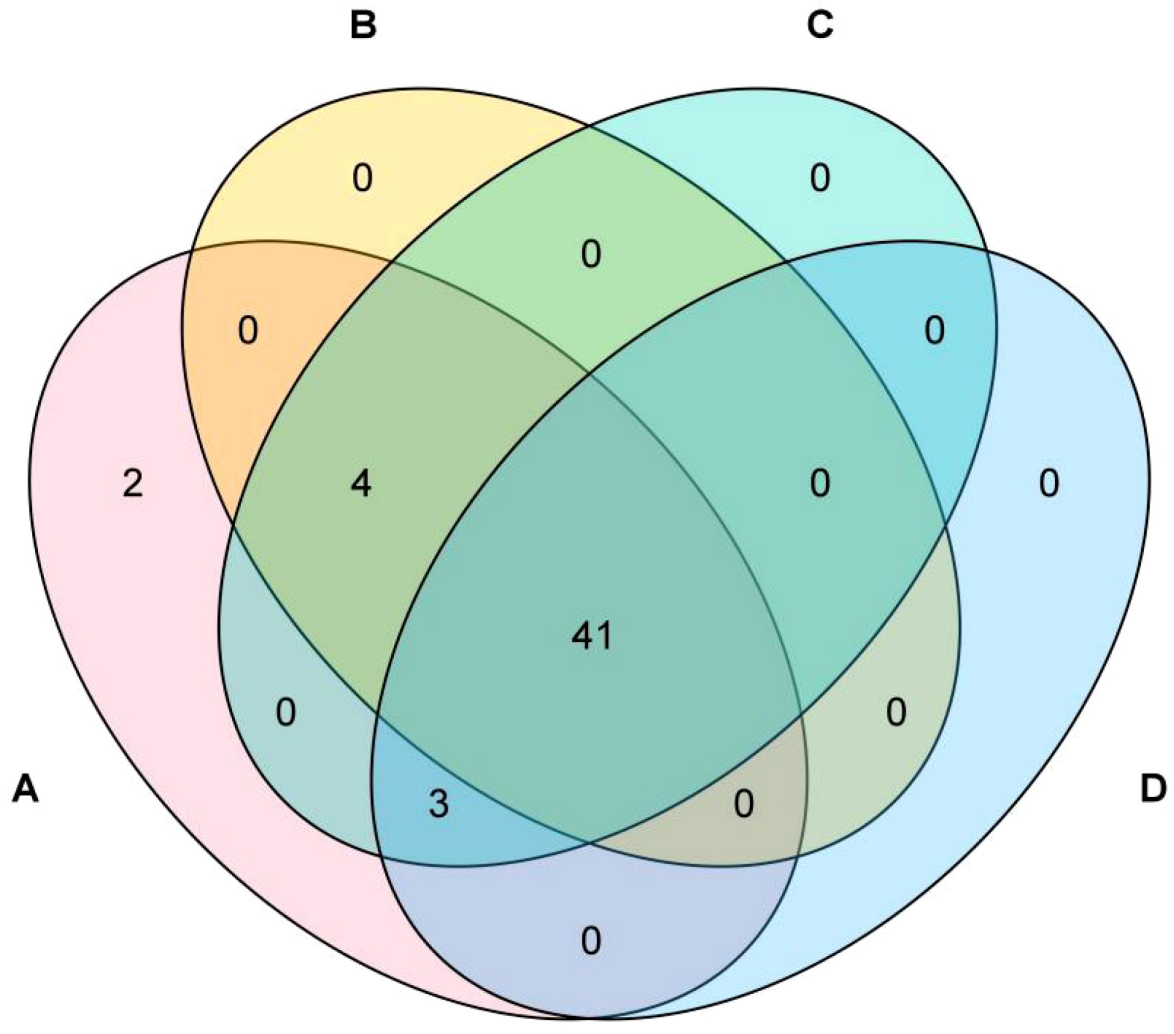
VENN [**(A)** formula 1; **(B)** formula 2; **(C)** formula 3; **(D)** the control group].

As demonstrated in **Figure 4**, metabolites with VIP values > 1 exhibit distinct expression patterns across experimental groups: Formula 2 showed significant upregulation of ethanol, N-hexanol, and 1-Octene-3-one compared to the control group (downregulated). Formula 1 displayed marked enrichment of Decanal, 1-Nonanol, 1-Hexadecene, Hexanal, and Methyl nonyl ketone, all suppressed in the control group. Formula 3 was characterized by elevated levels of Benzaldehyde, Phenylethanol, Octanol, and Oxime-, methoxy-phenyl-_ relative to the control group, where these metabolites remained at low abundance.

## 4 Discussion

This study explored the feasibility of using *Rosa roxbunghii* pomace as a substrate for cultivating mushrooms by setting up four different formulations and analyzing the growth, yield, and volatile flavor compounds. The results showed that formulation 2 and the control had the best mushroom yields and yield per bag, with 850g and 800g, respectively, significantly different from formulations 1 and 3. The moisture and ash contents of the mushrooms produced using the four different formulations all met the GB/T 38581-2020 standard (Liu et al., 2018). From an economic perspective, using formulation 2 as a substitute for sawdust partially alleviated the demand for sawdust while properly disposing of the RRP and reducing raw material costs.

There was little difference in the types of volatile compounds produced in mushrooms grown using the four ratios of RRP, which ranged from 44 to 50. The main types of compounds were aldehydes, alcohols, and ketones. However, there were significant differences in the concentrations of these compounds among formulations 1, 2, 3, and the control, indicating that the flavors were markedly different. Orthogonal Projections to Latent Structures Discriminant Analysis (OPLS-DA) modeling of 54 metabolites demonstrated a cumulative explanation of 94.3% of the X-matrix variance across orthogonal components (P1: 51.7%, P2: 22%, P3: 20.6%), with cross-validated predictive capability reaching 99.4%(Q²(cum)= 0.993). These results indicate that metabolic profile variations possess substantial discriminative power to differentiate experimental groups, underscoring the robustness and biological relevance of the model.

The relative content of octanol in the mushrooms produced using formulations 1, 2, 3, and the control were 66.68%, 66.57%, 64.67%, and 53.7%, respectively. The highest relative content of 1-octen-3-ol was found in formulation 2, at 29.16%, followed by formulation 1 at 19.49%, and the control had the lowest content at 9.14%, while formulation 3 had the lowest content at 6.12%. The relative content of sulfur-containing compounds was highest in the control, at 11.03%, followed by formulation 1 at 4.87%, formulation 3 at 6.22%, and formulation 2 at 3.36%. Principal component analysis showed that the volatile compounds produced in the four types of mushrooms were distributed in four quadrants, with formulation 2 and the control relatively far apart and located on the diagonal, indicating significant differences in the volatile flavor compounds between formulation 2 and the control. The main compounds in formulation 2 were 1-octen-3-ol, trans-2-octen-1-ol, trans-2-octenal, 2-methyl-1-butanol, 2-methyl-1-butanal, and 1-octene, while the main compounds in control were methanethiol, acetone, dimethyl disulfide, pentanal, dimethyl tetrasulfide, and pleurotin.

The addition of *Rosa roxbunghii* pomace (RRP) to the cultivation medium likely affects the nitrogen source, C/N ratio, and pH, which in turn influence the flavor of the cultivated mushrooms. RRP contains various components, such as woody fibers, and its addition may change the nutrient composition of the medium. For example, the nitrogen source provided by RRP could impact the synthesis of volatile compounds during mushroom growth. According to previous studies (Eras-Muñoz et al., 2022; Nandakumar et al., 2018), changes in the nitrogen source can lead to variations in the metabolic pathways of microorganisms, potentially altering the production of volatile flavor compounds.

Moreover, the C/N ratio is an important factor in mushroom cultivation. A suitable C/N ratio can promote the growth and metabolism of mycelium. The addition of RRP may adjust the C/N ratio of the medium, affecting the mycelium’s ability to synthesize and accumulate volatile compounds. Monga et al. (2022) demonstrated that an inappropriate C/N ratio can limit the production of specific volatile compounds in mushrooms.

In addition, the pH of the medium can also be affected by RRP. Different pH values can influence the activity of enzymes involved in aroma metabolism. Enzymes play a crucial role in the synthesis and degradation of volatile compounds. For instance, some enzymes are more active under specific pH conditions, which can produce different volatile compounds or changes in their relative contents (Espino-Díaz et al., 2016).

The metabolism of volatile compounds in mushrooms is a complex process. It involves multiple enzymatic reactions. The addition of RRP may activate or inhibit certain enzymes related to aroma metabolism. For example, enzymes responsible for the synthesis of 1-octen-3-ol, a key flavor compound in mushrooms, may be affected by the components in RRP. Future research could focus on identifying these enzymes and clarifying how RRP affects their activity to understand the flavor improvement mechanism further.

## 5 Conclusion

In conclusion, cultivating mushrooms using *Rosa roxbunghii* pomace is feasible, and this study has found a new way to utilize *R. roxbunghii* pomace. It has also identified a new potential substrate for mushroom cultivation. Replacing 30% of sawdust with RRP can lower costs while ensuring yields, and formulation 2 had the highest relative content of the characteristic compound, 1-octen-3-ol, at 29.16%.

From an industrial application perspective, this research provides a valuable reference for the edible fungus industry in Guizhou. By using RRP as a cultivation substrate, local farmers and enterprises can reduce reliance on traditional sawdust resources, which may face shortages in the long term. This not only helps to dispose of RRP in an environmentally friendly way but also cuts down on raw material costs, enhancing the overall economic efficiency of the industry. Additionally, the unique flavor of shiitake mushrooms cultivated with this substrate, especially the high content of 1 - octen - 3 - ol in formula 2, can potentially meet the diverse needs of consumers in the market, adding value to the product.

For future studies, exploring the application of *Rosa roxbunghii* pomace in cultivating other strains of Lentinula edodes would be interesting. Different strains may respond differently to the addition of *Rosa roxbunghii* pomace, which could lead to further optimization of cultivation formulas and improved yields or flavor profiles. Moreover, investigating the use of other agricultural - products in combination with *Rosa roxbunghii* pomace could open up new possibilities for substrate development. This could help to create more diverse and sustainable cultivation substrates, further promoting the development of the edible fungus industry.

## Data Availability

The original contributions presented in the study are included in the article/supplementary material. Further inquiries can be directed to the corresponding author.
